# Incident COVID-19 and Hospitalizations by Variant Era Among Vaccinated Solid Organ Transplant Recipients

**DOI:** 10.1001/jamanetworkopen.2023.29736

**Published:** 2023-08-18

**Authors:** Teresa Po-Yu Chiang, Aura T. Abedon, Jennifer L. Alejo, Dorry L. Segev, Allan B. Massie, William A. Werbel

**Affiliations:** 1 Department of Surgery, NYU Grossman School of Medicine, New York; 2 Department of Surgery, Johns Hopkins University School of Medicine, Baltimore, Maryland; 3 Department of Medicine, Johns Hopkins University School of Medicine, Baltimore, Maryland

## Abstract

This cohort study evaluates the incidence of COVID-19 and hospitalizations across variant eras in 2021 and 2022 among vaccinated solid organ transplant (SOT) recipients.

## Introduction

Solid organ transplant recipients experienced severe COVID-19 outcomes before vaccination and high breakthrough rates despite primary vaccine series.^1,2^ Infection risks among transplant recipients amid shifting immunity landscape and Omicron subvariant waves are not well described. Understanding longitudinal COVID-19 outcomes on a population scale has implications for future counseling and risk prediction; thus, we quantified changes in COVID-19 burden among transplant recipients, hypothesizing reduced disease severity in recent variant eras among SARS-CoV-2 vaccine uptake.

## Methods

Transplant recipients reporting 1 or more SARS-CoV-2 vaccination within a national prospective cohort were surveyed following immunoprophylaxis events to ascertain incident COVID-19 (self-reported positive molecular or antigen test result) and hospitalizations between January 2021 and December 2022, with additional cohortwide surveys in January and June 2022 and unsolicited reporting (eMethods in Supplement 1). Follow-up time accrued from first vaccination through last survey. This cohort study followed the STROBE reporting guideline and was approved by Johns Hopkins University, with waiver of documentation of informed consent because of no more than minimal risk intervention.

Cohort characteristics, COVID-19 incidence rate, and hospitalization ratio were reported by variant era: pre-Delta (January to May 2021), Delta (June to December 2021), and Omicron BA.1 (January to March 2022), BA.2 (April to June 2022), and BA.4-BA.5-BQ.1 (July to December 2022). Monthly transplant recipient COVID-19 incidence rate was plotted against US population case counts.^3^ Post hoc, COVID-19 hospitalization ratio and serious COVID-19 disease (hospitalizations plus deaths) were compared between post-BA.1 and pre-Delta, Delta, and BA.1 waves using Poisson regression. Two-sided *P* < .05 was significant. Stata/SE, version 17 was used for analysis.

## Results

Of 2461 participants, 2356 (95.7%) responded to 1 or more survey. Vaccinations increased over time (Table). COVID-19 survey response rate was 76.1% (13 483 of 17 726; median responses/participant, 5 [IQR, 3-7]); 464 of 2356 participants (19.7%) reported SARS-CoV-2 infection (15 reinfections). Among 936 330 person-days across all eras, COVID-19 incidence (per 1 000 000 person-days) was 90 for pre-Delta; 304, Delta; 1292, BA.1; 1051, BA.2; and 1066, BA.4-BA.5-BQ.1. Overall incidence was lower among participants 60 years or older vs younger than 60 years (420.2 vs 605.0; *P* < .001) and similar if less than 2 years vs 2 years or more from transplant (599.6 vs 485.7; *P* = .06) and in lung vs nonlung recipients (572.8 vs 500.4; *P* = .37). Transplant recipient COVID-19 incidence paralleled US population case counts (Figure, A), peaking during the BA.1 wave, albeit with greater relative decreases in US cases in later eras.

**Table.** Longitudinal Demographics, Transplant Factors, and COVID-19 Diagnoses Among Solid Organ Transplant Recipients in a National Prospective Cohort

Characteristic	2021, No. (%)		2022, No. (%)				Total, No. (%) (N = 2356)	COVID-19 hospitalization, No. (%)		*P* value^b^
	Jan (n = 129)	Jun (n = 2056)	Jan (n = 1829)	Apr (n = 977)	Jul (n = 835)	Dec (n = 156)		Yes (n = 37)	No (n = 430)^a^	
Age, median (IQR), y^c^	49 (38-59)	60 (48-68)	61 (50-69)	64 (54-70)	65 (55-70)	65 (57-69)	59 (47-67)	60 (50-67)	59 (47-68)	.43
Sex, No./total No. (%)										
Female^d^	76/123 (61.8)	1088/1981 (54.9)	973/1763 (55.2)	537/948 (56.6)	456/811 (56.2)	95/153 (62.1)	1228/2268 (54.1)	22/36 (61.1)	265/419 (63.2)	.86
Male	47/123 (38.2)	893/1981 (45.1)	790/1763 (44.8)	411/948 (43.4)	355/811 (43.8)	58/153 (37.9)	1040/2268 (45.9)	14/36 (38.9)	154/419 (36.8)	
Race, No./total No. (%)^e^										
Asian	9/122 (7.4)	78/1963 (4.0)	69/1746 (4.0)	40/939 (4.3)	36/802 (4.5)	13/149 (8.7)	97/2249 (4.3)	0/36	12/415 (2.9)	.95
Black or African American	2/122 (1.6)	61/1963 (3.1)	49/1746 (2.8)	22/939 (2.3)	16/802 (2.0)	4/149 (2.7)	75/2249 (3.3)	1/36 (2.8)	17/415 (4.1)	
White	107/122 (87.7)	1768/1963 (90.1)	1578/1746 (90.4)	854/939 (90.9)	731/802 (91.1)	129/149 (86.6)	2004/2249 (89.1)	34/36 (94.4)	373/415 (89.9)	
Other^f^	4/122 (3.3)	56/1963 (2.9)	50/1746 (2.9)	23/939 (2.4)	19/802 (2.4)	3/149 (2.0)	73/2249 (3.2)	1/36 (2.8)	13/415 (3.1)	
Organ type										
Kidney	69 (53.5)	1048 (51.0)	951 (52.0)	507 (51.9)	434 (52.0)	76 (48.7)	1184 (50.3)	19 (51.4)	211 (49.1)	.003
Liver	20 (15.5)	436 (21.2)	385 (21.0)	194 (19.9)	159 (19.0)	36 (23.1)	504 (21.4)	1 (2.7)	107 (24.9)	
Pancreas	4 (3.1)	16 (0.8)	12 (0.7)	5 (0.5)	5 (0.6)	2 (1.3)	22 (0.9)	0	4 (0.9)	
Lung	7 (5.4)	169 (8.2)	148 (8.1)	90 (9.2)	77 (9.2)	15 (9.6)	195 (8.3)	9 (24.3)	40 (9.3)	
Heart	19 (14.7)	232 (11.3)	201 (11.0)	119 (12.2)	105 (12.6)	18 (11.5)	276 (11.7)	5 (13.5)	40 (9.3)	
Multiorgan, intestine, or VCA	10 (7.8)	155 (7.5)	132 (7.2)	62 (6.3)	55 (6.6)	9 (5.8)	175 (7.4)	3 (8.1)	28 (6.5)	
Time since transplant										
<1 y	12 (9.3)	112 (5.4)	12 (0.7)	0	0	0	203 (8.6)	2 (5.4)	4 (0.9)	.11
1-5 y	48 (37.2)	720 (35.0)	653 (35.7)	356 (36.4)	295 (35.3)	47 (30.1)	790 (33.5)	14 (37.8)	163 (37.9)	
>5 y	69 (53.5)	1224 (59.5)	1164 (63.6)	621 (63.6)	540 (64.7)	109 (69.9)	1363 (57.9)	21 (56.8)	263 (61.2)	
Immunosuppression										
CNI	121 (93.8)	1773 (86.2)	1568 (85.7)	838 (85.8)	723 (86.6)	132 (84.6)	2034 (86.3)	30 (81.1)	383 (89.1)	.17
mTOR inhibitor	12 (9.3)	254 (12.4)	225 (12.3)	137 (14.0)	118 (14.1)	29 (18.6)	289 (12.3)	5 (13.5)	49 (11.4)	.60
Mycophenolate	91 (70.5)	1354 (65.9)	1214 (66.4)	679 (69.5)	592 (70.9)	99 (63.5)	1518 (64.4)	31 (83.8)	283 (65.8)	.03
Corticosteroid	65 (50.4)	1116 (54.3)	985 (53.9)	534 (54.7)	463 (55.4)	87 (55.8)	1283 (54.5)	28 (75.7)	237 (55.1)	.02
Belatacept	0	55 (2.7)	52 (2.8)	36 (3.7)	30 (3.6)	4 (2.6)	62 (2.6)	2 (5.4)	14 (3.3)	.37
3 Drugs^g^	47 (36.4)	744 (36.2)	664 (36.3)	370 (37.9)	329 (39.4)	59 (37.8)	837 (35.5)	21 (56.8)	157 (36.5)	.02
Vaccine doses^h^										
1	128 (99.2)	99 (4.8)	57 (3.1)	9 (0.9)	5 (0.6)	0	NA	NA	NA	<.001
2	1 (0.8)	1835 (89.3)	219 (12.0)	6 (0.6)	2 (0.2)	1 (0.6)	NA	NA	NA	
3	0	101 (4.9)	1355 (74.1)	272 (27.8)	131 (15.7)	12 (7.7)	NA	NA	NA	
4	0	19 (0.9)	158 (8.6)	648 (66.3)	510 (61.1)	34 (21.8)	NA	NA	NA	
≥5	0	2 (0.1)	40 (2.2)	42 (4.3)	187 (22.4)	109 (69.9)	NA	NA	NA	

Abbreviations: CNI, calcineurin inhibitor; NA, not applicable; VCA, vascularized composite allografts.

^a^ Excludes 12 infection episodes without specified hospitalization status.

^b^ Wilcoxon rank-sum testing was used for continuous variables and Fisher exact testing for categorical variables.

^c^ Eleven participants did not report age.

^d^ Eighty-eight participants did not report sex.

^e^ Self-reported (107 did not report). Collected due to potential differences in SARS-CoV-2 exposure risks and receipt of care associated with sociodemographic factors that may affect infection and hospitalization rates.

^f^ American Indian or Alaska Native, Arab or Middle Eastern, Native Hawaiian or Other Pacific Islander, and multiracial.

^g^ Combination of antimetabolite, calcineurin inhibitor or mTOR inhibitor, and corticosteroid.

^h^ Median number of doses before COVID-19 diagnosis was 3 (IQR, 3-3) among hospitalized participants and 3 (IQR, 3-4) among nonhospitalized participants.

**Figure.**
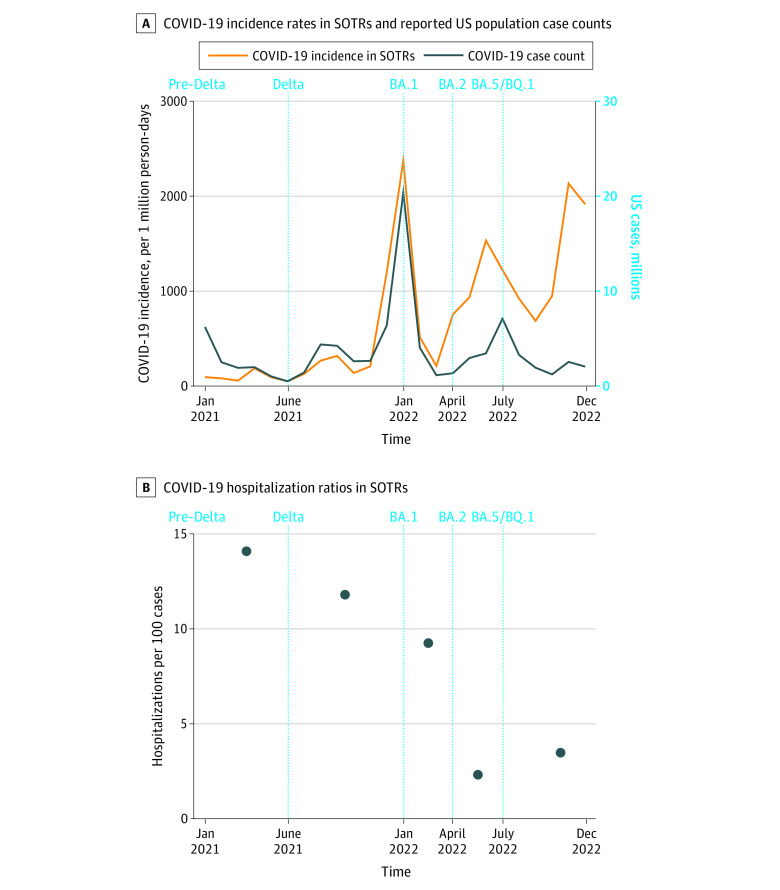
Trends in COVID-19 Incidence and Hospitalization Rates in a National Cohort of Vaccinated Solid Organ Transplant Recipients (SOTRs) Vertical dashed lines denote major SARS-CoV-2 variant eras per Centers for Disease Control and Prevention data.

There were 37 COVID-19-related hospitalizations among 35 of 464 participants (7.5%). Hospitalized participants more often reported stronger immunosuppression (eg, 3-drug regimens: 56.8% vs 36.5%; *P* = .02) and lung transplant (24.3% vs 9.5%; *P* = .01). Hospitalization ratios (per 100 incident infections) were 14.1 for pre-Delta; 11.8, Delta; 9.2; BA.1; 2.3, BA.2; and 3.5, BA.4-BA.5-BQ.1 (Figure, B). Overall hospitalization ratio was similar among participants 60 years or older vs younger than 60 years (8.3 vs 7.4; *P* = .75) and less than 2 years vs 2 years or more from transplant (8.6 vs 7.6; *P* = .74) yet higher in lung vs nonlung recipients (18.4 vs 6.6; *P* = .007).

Hospitalization ratio comparing post-BA.1 vs pre-Delta, Delta, and BA.1 was 0.27 (95% CI, 0.11-0.69; *P* = .006), with a similar ratio of serious COVID-19 disease (0.29; 95% CI, 0.12-0.69; *P* = .005). Of 16 reported deaths, 5 were COVID-19 related (1 post-BA.1); 6, non-COVID-19 related (3 post-BA.1); and 5, unknown cause (4 post-BA.1).

## Discussion

In this study, 19.7% of participants reported incident COVID-19, 7.5% of whom required hospitalization. Transplant recipient COVID-19 incidence followed US case count trends, with higher incidence in later Omicron waves potentially due to home test capture and/or increased susceptibility to SARS-CoV-2 variants.

COVID-19-related hospitalization among transplant recipients markedly decreased after the BA.1 wave, likely owing to changing population immunity,^4^ improved therapeutics,^5^ home testing access, and/or differing viral pathogenicity.^6^ Limitations include sampling and recall bias due to observational study design, lack of SARS-CoV-2 sequencing or longitudinal testing frequency, and incomplete death ascertainment.

## Notes

Supplement 1.
eMethods.

Supplement 2.
Data Sharing Statement
