# Supplementary material for: Incident COVID-19 and Hospitalizations by Variant Era Among Vaccinated Solid Organ Transplant Recipients
Source: JAMA Netw Open. 2023 Aug 18;6(8):e2329736. doi: 10.1001/jamanetworkopen.2023.29736 (PMC10439474; doi:10.1001/jamanetworkopen.2023.29736)
Supplement: Supplement 1. — eMethods. [file jamanetwopen-e2329736-s001.pdf]

Supplemental Online Content

Chiang TPY, Abedon AT, Alejo JL, Segev DL, Massie AB, Werbel WA. Incident COVID-19 and hospitalizations by variant era among vaccinated solid organ transplant recipients. *JAMA Netw Open.* 2023;6(8):e2329736. doi:10.1001/jamanetworkopen.2023.29736

eMethods.

This supplemental material has been provided by the authors to give readers additional information about their work.

© 2023 Chiang TPY et al. *JAMA Network Open.*

eMethods

<u>Reporting of COVID-19 cases and calculation of incidence rates in solid organ transplant recipients</u>

Within a national prospective, observational cohort (Johns Hopkins University IRB# 00248540), solid organ transplant recipients (SOTRs) reported baseline history including prior COVID-19 on enrollment. Incident COVID-19 diagnoses and the date of positive testing were reported on serial digital surveys delivered (i) 1-week after receiving each SARS-CoV-2 vaccination or monoclonal antibody prophylaxis (tixagevimab and cilgavimab, T+C), (ii) 3-months following completion of the second COVID-19 vaccine, or the second dose of T+C, and (iii) augmented by additional cohort-wide surveys on January 28, 2022, and June 17, 2022. Unsolicited event reporting was also continuously recorded by the study team. For participants who reported ≥2 incident COVID-19 tests during follow up, these were counted as discrete episodes if occurring ≥60 days apart (otherwise the date of first positive test was used).

Monthly COVID-19 incidence rate was derived by dividing the monthly total case counts across the cohort by the monthly total contributed person-days during that period, and reported as cases per 1,000,000 person-days. Person-time was contributed beginning at date of first SARS-CoV-2 vaccination. If a specific date was not reported for an incident case (n=24 [5%] of cases, none hospitalized), this case was “distributed” across the period of time elapsing between the prior and incident survey dates. For example, an incident, undated case reported between two surveys dated 1/22/2022 and 2/20/2022 would contribute 1/3 of a case to the month of January and 2/3 of a case to the month of February.

<u>Calculation of COVID-19 hospitalization ratio among SOTRs, by SARS-CoV-2 variant era</u>

On the same longitudinal survey instruments, SOTRs were asked whether their COVID-19 diagnosis was associated with hospitalization and the date of admission. COVID-19 hospitalization ratio by SARS-CoV-2 variant era was derived by dividing number of reported hospitalizations by the total case counts

© 2023 Chiang TPY et al. *JAMA Network Open.*

accumulated during each period, and reported as hospitalizations per 100 cases. Variant era time bins were designated on the month scale, spanning from the first through the last month a reported variant was considered dominant (i.e., represented the highest proportion of sequenced variants per weighted or smoothed CDC surveillance samples for the >2 weeks of the month) using CDC variant surveillance data.[1,2] Ultimately, eras were designated pre-Delta: 01/2021-05/2021; Delta: 06/2021-12/2021; Omicron BA.1-BA.1.1: 01/2022-03/2022; BA.2-BA.2.12.1: 04/2022-06/2022; BA.4-BA.5-BQ.1: 07/2022-12/2022. After plotting hospitalization ratios over time, post-hoc Poisson regression was utilized to test whether the proportion of hospitalizations among cases differed in the early variant/BA.1 era versus the post-BA.1 era (i.e., over the BA.2, BA.4/5, and BQ.1 predominant waves). Sensitivity analyses excluded 12 (2.6%) infected participants without valid survey responses for hospitalization.

Death Ascertainment

In contrast to incident COVID-19 diagnoses or hospitalizations, voluntary death reporting was provided by close household contacts to the study team via secure email communication. Exact date and circumstances of death were not always reported by the contacts and were therefore not always available. Of 16 reported deaths, 14 had a specified death date or ascertainable variant era, and 11 had a specified cause of death (categorized into COVID-19-related, non-COVID-19-related); no participant was missing both date and cause of death. Sensitivity analysis utilized a conservative composite outcome of serious COVID-19 disease (COVID-19-related hospitalization plus COVID-19-related deaths plus deaths of unknown cause).

© 2023 Chiang TPY et al. *JAMA Network Open.*

Supplementary methods references:

1. Centers for Disease Control and Prevention, COVID-19 Response. COVID-19 Case Surveillance Public Use Data with Geography (version date : January 12, 2022).
2. Lambrou AS, Shirk P, Steele MK, et al. Genomic Surveillance for SARS-CoV-2 Variants : Predominance of the Delta (B.1.617.2) and Omicron (B.1.1.529) Variants — United States, June 2021–January 2022. MMWR Morb Mortal Wkly Rep 2022 ;71 :206–211. DOI : http://dx.doi.org/10.15585/mmwr.mm7106a4

© 2023 Chiang TPY et al. *JAMA Network Open.*
