# Supplementary material for: Incident COVID-19 and Hospitalizations by Variant Era Among Vaccinated Solid Organ Transplant Recipients
Source: JAMA Netw Open. 2023 Aug 18;6(8):e2329736. doi: 10.1001/jamanetworkopen.2023.29736 (PMC10439474; doi:10.1001/jamanetworkopen.2023.29736)
Supplement: Supplement 2. — Data Sharing Statement [file jamanetwopen-e2329736-s002.pdf]

Data Sharing Statement

Chiang. Incident COVID-19 and Hospitalizations by Variant Era Among Vaccinated Solid Organ Transplant Recipients. *JAMA Netw Open*. Published August 18, 2023. doi:10.1001/jamanetworkopen.2023.29736

Data Yes
Data available: Deidentified participant data. Requests for deidentified data may be sent to Dr. William Werbel (PI, wwerbel1@jhmi.edu) and the Johns Hopkins Transplant Research Center (TRCadministration@jh.edu) for review. Data requests will also require review by the Johns Hopkins Institutional Review Board for consideration of a data use agreement.
